# Physiologically based mathematical models of nanomaterials for regulatory toxicology: A review

**DOI:** 10.1016/j.comtox.2018.10.002

**Published:** 2019-02

**Authors:** L. Lamon, D. Asturiol, A. Vilchez, J. Cabellos, J. Damásio, G. Janer, A. Richarz, A. Worth

**Affiliations:** a European Commission, Joint Research Centre, Ispra (VA), Italy; b Leitat Technological Center, c/de la Innovació 2, Terrassa, Barcelona, Spain

**Keywords:** Toxicokinetics, Physiologically based model, Manufactured nanomaterial, REACH, Regulatory acceptance

## Abstract

The development of physiologically based (PB) models to support safety assessments in the field of nanotechnology has grown steadily during the last decade. This review reports on the availability of PB models for toxicokinetic (TK) and toxicodynamic (TD) processes, including *in vitro* and *in vivo* dosimetry models applied to manufactured nanomaterials (MNs). In addition to reporting on the state-of-the-art in the scientific literature concerning the availability of physiologically based kinetic (PBK) models, we evaluate their relevance for regulatory applications, mainly considering the EU REACH regulation. First, we performed a literature search to identify all available PBK models. Then, we systematically reported the content of the identified papers in a tailored template to build a consistent inventory, thereby supporting model comparison. We also described model availability for physiologically based dynamic (PBD) and *in vitro* and *in vivo* dosimetry models according to the same template. For completeness, a number of classical toxicokinetic (CTK) models were also included in the inventory. The review describes the PBK model landscape applied to MNs on the basis of the type of MNs covered by the models, their stated applicability domain, the type of (nano-specific) inputs required, and the type of outputs generated. We identify the main assumptions made during model development that may influence the uncertainty in the final assessment, and we assess the REACH relevance of the available models within each model category. Finally, we compare the state of PB model acceptance for chemicals and for MNs. In general, PB model acceptance is limited by the absence of standardised reporting formats, psychological factors such as the complexity of the models, and technical considerations such as lack of blood:tissue partitioning data for model calibration/validation.

## Nomenclature

ADME: adsorption, distribution, metabolism and excretion
ADSRM: agglomeration-diffusion-sedimentation-reaction model
AF: assessment factor
CFD: computational fluid dynamics
CTK: classical toxicokinetic
DNEL: derived no-effect level
ECHA: European Chemicals Agency
EURL ECVAM: European Union Reference Laboratory for Alternatives to Animal Testing
GITT: generalised integral transform technique
GRACIOUS: grouping, read-across, characterisation and classification framework for regulatory risk assessment of manufactured nanomaterials and safer design of nano-enabled products
ISDD: *In vitro* sedimentation, diffusion and dosimetry model
IVIVE: *In vitro* to *in vivo* extrapolation
MN: manufactured nanomaterial
MPPD: multiple-path particle dosimetry
MPS: mononuclear phagocyte system
NAM: new approach methodology
PAMAM: polyamidoamine
PB: physiologically based
PBD: physiologically based dynamic
PBK: physiologically based kinetic
PEG: poly-ethylene glycol (PEG)
PVP: poly-vinyl pyrrolidone
QSAR: quantitative structure-activity relationship
REACH: registration, evaluation, authorisation and restriction of chemicals
RES: reticuloendothelial system
TD: toxicodynamics
TK: toxicokinetics

## 1 Introduction

Following exposure to a chemical or a manufactured nanomaterial (MN), a toxic response may occur, depending on the ability of the substance to reach the target tissue, and its intrinsic potency when interacting with that tissue. Toxicokinetics (TK) is the science that studies the fate of a substance (including MNs) in the body, whereas toxicodynamics (TD) studies what a substance does to the body once it comes to contact with it (local toxicity) or once it enters the body (systemic toxicity) [1].

Mathematical models that can predict the target tissue concentration of the toxicant or of its active species (parent compound or metabolite) are especially useful in chemical risk assessment; these models are termed physiologically based kinetic (PBK) models.^1^ PBK models aim at the development of quantitative descriptions of adsorption, distribution, metabolism and excretion (ADME) of chemicals, on the basis of interrelationships among the critical determinants of these processes.

PBK model parameters include physiological parameters, such as tissue volumes and physiological flow rates, along with chemical-specific parameters, such as rates of absorption, diffusion across cell membranes, blood:tissue partition coefficients, and rates/affinities for biochemical reactions and transporters. PBK models have traditionally been used for extrapolating: a) the kinetic behaviour of chemicals from test animal species to humans; b) one human exposure route to another; c) from high dose to low dose; and d) between internal and external exposure levels in the context of biological monitoring [2]. More recently, PBK models have also been used to improve the relevance of *in vitro* toxicity data by enabling *in vitro* to *in vivo* extrapolation (IVIVE), and the ADME parameters of such models are increasingly derived from new approach methodologies (NAMs) such as *in vitro* and *in silico* methods [3]. PBD models extend PBK models by including a model of the toxicodynamic response, typically by including an internal concentration-response relationship. An overview of available PBK models for chemicals, and of PBK platforms, is available in Bessems et al. [4].

In the European Union (EU), the REACH (Registration, Evaluation, Authorisation and Restriction of Chemicals) Regulation aims to improve the protection of human health and the environment from the risks that can be posed by chemicals, while enhancing the competitiveness of the EU chemicals industry. It also reduces animal testing by promoting the use of alternative methods in the hazard assessment of substances.

REACH applies to a wide range of chemical substances and manufactured nanomaterials (MNs), not only those used in industrial processes, but also those in our day-to-day lives, for example in cleaning products, paints as well as in articles such as clothes, furniture and electrical appliances.

To comply with REACH, companies must identify and manage the risks linked to the substances they manufacture and market in the EU. Companies are required to demonstrate how the substance can be safely used, and they must communicate the risk management measures to the users. If the risks cannot be managed, authorities can restrict the use of substances in different ways

For the purposes of REACH, MNs are defined by the European Commission *Recommendation on the Definition of a Nanomaterial* as materials containing particles, in an unbound state or as an aggregate or as an agglomerate and where, for 50% or more of the particles in the number size distribution, one or more external dimensions is in the size range 1 nm–100 nm [5].

PBK models are also taken in consideration in the REACH regulation as relevant in human health risk assessment according to the Chapter R8 of the Guidance on information requirements and chemical safety assessment on the characterisation of dose[concentration]-response for human health [6] where it is recognised that PBK modelling can support the derivation of derived no-effect level (DNEL) from animal data to account for human health risk. PBK models can be used to determine or adjust specific assessment factors (AFs): 1) route-to-route; 2) interspecies and 3) high-dose-low-dose extrapolation. In addition, PBK modelling data can aid in the quantification of intraspecies variability, denoted by variation in anatomical, physiological and biochemical parameters with age, gender, genetic predisposition and health status. With a view to replacing animal testing, PBK models can in principle also be used to extrapolate from effects observed in *in vitro* systems to the *in vivo* situation. TD models are also relevant in determining interspecies AFs, by taking into account the different susceptibility of the test animal compared to the human [7].

Although PBK models are considered in ECHA guidance supporting information requirements in chemical safety regulation [6], there is no official template to report the details of the PB models used or any set of criteria/rules to consider a given PBK or fate model as valid or adequate for a given purpose. In practice, the validity of a model, and the adequacy of its results, are evaluated on a case-by-case basis.

MNs are differing in composition, size, surface chemistry, morphology, and assessing each MN would require considerable technical and financial efforts [8]: for this reason, the understanding of MN pharmacokinetics has been addressed more and more in the scientific literature [9], [10], [11], and several PB models became available [12], [13], [14], [15], [16], [17].

In this paper, we treat PB models which include physiologically based kinetic (PBK) and dynamic (PBD) models, as well as *in vitro* and *in vivo* dosimetry models. For completeness, our model inventory includes several classical toxicokinetic (CTK) models, even though these are not physiologically based. Lamon et al. [18] developed a template for reporting PB models applied to MNs. This template was then used to compile the information on the available PB models applied to MNs. The original inventory is available in the public online inventory available at the JRC Science Hub [19] and the contents are presented in this review paper.

The objective of this paper is to report the status of PB and CTK models developed or applied to MNs, and to highlight the relevance of the existing models for the REACH regulation.

## 2 Methods

### 2.1 Bibliographic searches

The literature search aimed at collecting available models including PBK, PBD and dosimetry models either considering *in vivo* or *in vitro* systems. Searches were performed in Scopus and Web of Science.

A specific search on *ATLA* (*Alternatives to Laboratory Animals*) in Pubmed was also conducted given that this journal is not considered within the Scopus database. We selected 50 publications as relevant to feed the model inventory. In some cases, multiple papers focused on different applications of the same model. These papers have not been included in the inventory as new entries, but as different applications of the same model. After identifying this kind of publication, and exclusion of redundant models, 35 papers were included in the final inventory.

More details on the bibliographic searches are available in Worth et al. [20]. Papers published until 2016 are reported in the public online inventory available at the JRC Science Hub [19]. An update on the available PB models in the literature was carried out in May 2018, but this search did not identify new models.

### 2.2 Model types considered in this review

PB models available in the literature were organised as follows:

1. Physiologically based toxicokinetic (TK) models: Numerical models commonly derived from physiologically relevant compartments and processes (PBK models) and constructed from mass-balance equations (i.e. accounting for material entering and leaving a system). CTK models are also included within this category.
2. Physiologically based toxicodynamic (TD) models, i.e. PBD models: Models that simulate the intensity and time-course of effects caused by a MN on a biological system (e.g. prediction of the inflammatory response of macrophages under exposure to MNs).
3. Dosimetry models: Computational models that predict the fate and the local concentration/dose of MNs in a defined *in vitro* or *in vivo* system. The models in this section are divided in two different categories:
  3.1. Respiratory tract dosimetry: biologically-based mechanistic approaches to predict the fate of inhaled particles, by describing the physical and physiological factors that influence the deposition, clearance, and retention of inhaled particles.
  3.2. *In vitro* dosimetry: models that calculate the dose-rates and target cell doses based on particle kinetics and transport prediction of MNs to cells in liquid-based *in vitro* systems.

In the next section, we provide the state of the art in the different model domains according to the information collected in the inventory.

### 2.3 The model inventory

A detailed description of the structure of the model inventory is available in Lamon et al. [18]. Information on the available models cover different domains:

• Model metadata: includes model details (name, version, homepage) information about the model owner (ownership, contact point, email address, license), the reference (associated literature references and DOI). A model name was assigned taking into consideration the aim and the type of model.
• Model description: this section gathers the main characteristics of the model (i.e. a generic description of the model output(s), the level of organisation considered (i.e. compartments, tissues, cells), the model type), information about the processes considered within the model (including units, level of description/definition). This section also allows the possibility to include free text to add comments.
• Inputs and Outputs: Information on the nano-specific or chemical-specific parameters that the model uses as input or output is reported in this section (e.g. parameter, symbol, units, protocol for measured values, etc). Assumptions or key information on the protocol related to the inputs are also covered by this section.
• NP description: in this section the type of MNs used to build the model or to evaluate it are described (e.g. TiO_2_, Ag, CeO_2_, metal oxide, carbon-based, polystyrene, etc.). Then other associated physicochemical properties, such as coating, size, shape, and any other relevant characterisation performed. This also contains the description of the MNs used in other literature references evaluating or using the same model. The information about these references is placed in the subsection “Used in reference”.
• Model domain: in this section the applicability domain stated by the author or inferred from the description and the outcome(s) of the model is provided. It also state if one or more physicochemical properties of the MNs (e.g. size, density, agglomeration state, etc.) are used as input model parameters. General adopted assumptions by the model are also described at this level.

We have populated the inventory reported in Lamon et al. [18] with the models available in the literature, collected through the bibliographic search described above. The resulting inventory is freely available through the JRC Science Hub [21] and through the European Union Reference Laboratory for Alternatives to Animal Testing (EURL-ECVAM) collection [19] in the JRC Data catalogue as .xls files^2^.

## 3 Results on available PB models applicable to MNs

In this section, we report the contents of the inventory, to describe the status of PB models developed or applied to MNs.

**Fig. 1:**
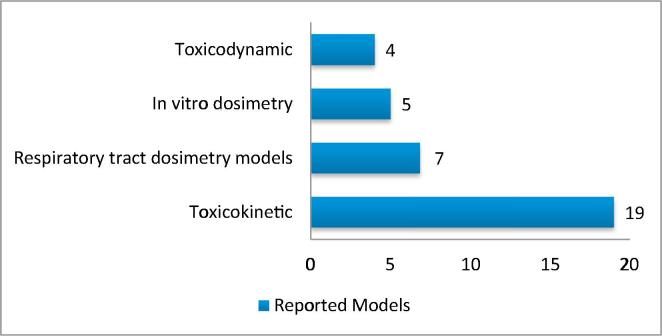
Number of publications of each model included in the Model Inventory [19].

Fig. 1 shows the number of papers reported in the inventory for each model type.

**Fig. 2:**
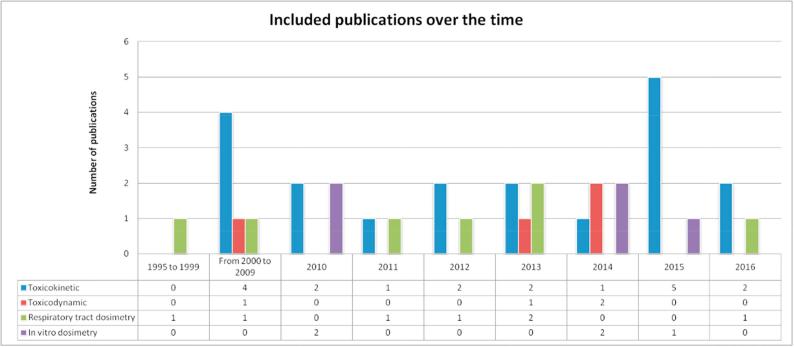
Number of publications included in the Model Inventory [19] by publication year.

In addition, Fig. 2 depicts the number of publications included in the inventory with respect to the year of publication. Based on this figure, the number of PB publications considered as relevant remains almost constant over the time. A peak of PB publications (n = 5) was observed in 2015. A constant number of publications is also observed for the respiratory tract dosimetry models. For the remaining models there is some variability depending on the year of publication. It should be noted that this figure reflects the criteria adopted to include the publications as “relevant models” (principally new developed models) but it does not reflect the actual number of publications using these models in the field of nanotoxicology.

### 3.1 Results of the analysis of the available TK models

**Fig. 3:**
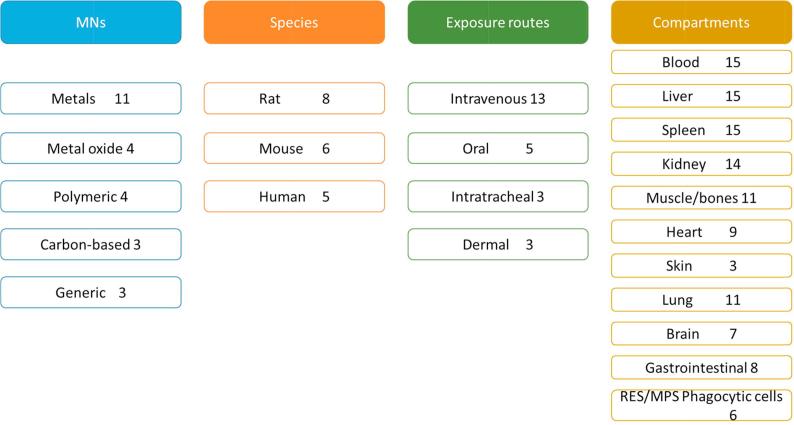
Summary of the type of MN, animal species, exposure routes and compartments used in the TK models for MNs. Information derived from [9], [12], [14], [17], [28], [29], [30], [31], [32], [33], [34], [35], [36], [37], [22], [23], [24], [25], [26], [27]. The numbers represent the times that each specific parameter has been identified. MPS: mononuclear phagocyte system; RES: reticuloendothelial system.

The parameters considered most relevant for the characterisation of a TK model are summarised in Fig. 3. These include: the type of MN used either to develop or evaluate the model, the animal species, the exposure routes, and the physiological compartments (organs or tissues) considered.

The TK models reported in the inventory cover a total of 15 different MNs including metals, metal oxides, polymeric and carbon-based nanomaterials. Metal based MNs are the most common materials (covered by 10 out of 19 models). As seen in Table 1, Ag (4) and Au (3) are the most frequently evaluated metals. Metal oxides, polymeric and carbon-based nanomaterials are represented to a similar extent. Three of the models [25], [34], [35] are not focused on the evaluation of a specific type of MN. They can be applied to MNs in a broad, generic sense, independently of their chemical composition. Table 1 shows a list with the specific type of MN for each group.

**Table 1:** List of MNs reported in the Inventory for TK models. The number in brackets indicates the how many models are available on each MN, including both publications on TK and CTK models.

Type of MNs	Specific type of MNs
Metal	Silver (4), Gold (3), CdSeTe (1), Iridium (1), CdTe (1), CdS (1)
Metal oxide	Fe_2_O_3_ (1), TiO_2_ (1), ZnO (1), SiO_2_ (1)
Polymeric	Polystyrene (1), Poly(amidoamine(1), PLGA (1), polyacrylamide (1)
Carbon-based	Carbon (3)
Unspecified chemical composition	(3)

Six models [14], [24], [38], [34], [35], [36] are considered as CTK, since they were based on the use of compartments without physiological meaning to calculate absorption, transportation, and elimination rate constants. Despite their limited capability to extrapolate between species or exposure routes, they can be useful to extrapolate between dose levels.

Fig. 3 shows that the intravenous route is the most common route of administration taken into consideration in developing TK models. Other administration routes such as those relevant for the oral route, the inhalation route (e.g. intratracheal administration), the dermal or the subcutaneous route are represented at a lesser extent in the inventory.

The TK models compiled within the inventory vary in complexity from full TK models where organs and tissues are considered as separate perfused compartments (i.e. Péry et al. [33] included more than 20 compartments) to more simplified, minimal TK models (i.e. van Kesteren et al. [14] included three compartments). Blood, liver, spleen and kidneys are the most represented compartments (see Fig. 3). Liver and spleen are considered the major target organs for the accumulation of MNs, especially after intravenous administration and together with blood, the main compartments containing reticuloendothelial system (RES; also called mononuclear phagocyte system or MPS) cells. MNs are rapidly captured and retained by these cell types (monocytes circulating in the blood, reticular cells in the spleen and Kupffer cells in the liver). The RES has been considered as a separate compartment in six models [22], [27], [30], [34], [39], [40].

### 3.2 Results of the analysis of available TD models

TD refers to the quantitative description of a toxicant’s effects on a biological system at different levels (from the molecular level, to higher levels such as cells, tissues, organ systems). In the model inventory, four TD models are reported; some details are extracted in Table 2.

**Table 2:** List of the details of selected TD models reported.

MN (size)	Endpoint	Biological level	Species	Reference
nano-Al (80 nm)	Functional viability	Alveolar macrophages cell dynamics	Rat	[41]
polyamidoamine dendrimer (PAMAM)	Uptake/cytotoxicity	(sub-)Cellular responses (e.g. ROS, apoptosis) for human keratinocyte and murine macrophages	Human Murine	[42]
nano-Ag carbon black (10–20 nm)	Pulmonary function	Alveolar tissue	Mouse	[43]
nano-Ag citrate-coated and PVP-coated	*In vitro* inflammatory response	Immune cell – macrophages and human monocyte-derived macrophages for cytokine study	Human	[44]

Shelley et al. [41] developed a model to simulate the cell population dynamics (including toxic effects and functional viability along time) of rat alveolar macrophages under exposure to nano-Al (size: 80 nm). A system of differential equations was derived based on the macrophage population, MNs concentration, and macrophage phagocytosis function. The model demonstrates how *in vivo* cell dynamics can be simulated starting from *in vitro* data.

Maher et al. [42] used a phenomenological rate equation model that numerically simulates uptake and cellular responses to polyamidoamine dendrimer (PAMAM) MN with a different number of initial branching points. The model simulates MN uptake and the subsequent cellular response measured by change in cellular markers of oxidative stress, mitochondrial damage, inflammatory response and apoptosis. The model is intended to be applied as a tool to interpolate and visualise the dose range and to elucidate the mechanisms underlying the *in vitro* cytotoxic response to MN exposure in time.

Mukherjee et al. [43] developed a multiscale TD model to quantify and predict pulmonary effects due to uptake of MNs in mice. The kinetics of surfactant and pulmonary function due to interactions of MNs at the alveolar microenvironment are simulated. This consists of a collection of TD modules to describe the dynamics of tissue focused on cells and the alveolar surfactant chemicals that regulate the process of breathing, as well as the response of the pulmonary system to xenobiotics. It is worth mentioning that the model uses some size-related (e.g. diameter and surface area) or surface-related properties (e.g. zeta potential) as input parameters. The model predictions were compared to *in vivo* lung function response in mice and the analysis of mice lung lavage fluid following exposures to citrate-stabilised 10–20 nm nano-Ag and carbon black as MNs.

Finally, Mukherjee et al. [44] developed a mathematical model that predicts the *in vitro* inflammatory response (i.e. expression levels of cytokines) of immune cells exposed to citrate-coated and PVP-coated nano-Ag in a culture system. The model was run with and without the inclusion of the NP agglomeration-diffusion-sedimentation-reaction model [45], to determine the extent of effects due to *in vitro* cellular dosimetry of MNs.

The available TD models focus on cellular, sub-cellular or tissue levels. Two out of four models focus on alveolar cells or tissue [41], [43], one is developed for macrophages [44] and one for different cell lines [42].

### 3.3 Results of the analysis of available respiratory tract dosimetry models

The respiratory tract dosimetry models compiled in this section are based on modelling fluid and particle dynamics in subject-specific respiratory geometry tracts. Among these modelling techniques, computational fluid dynamics (CFD) models allow for simulations of airflow patterns and particle deposition efficiencies in complex geometries such as those found in the upper respiratory tract of laboratory animals and humans. These models provide a valuable supplement to experimental work in evaluating dose-response relationships.

**Fig. 4:**
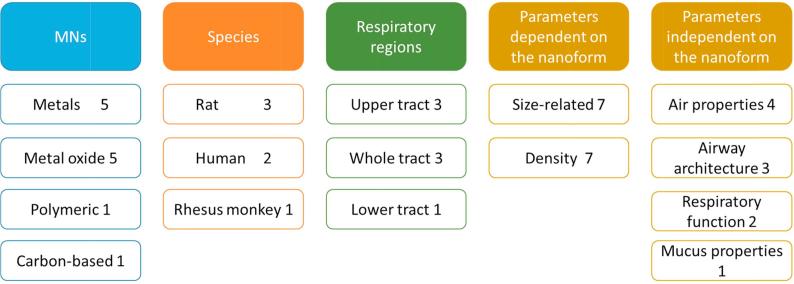
Summary of the type of MN, species/system, and input parameters (dependent or independent on the nanoform) used in the respiratory tract dosimetry models. The numbers represent the times that each specific parameter was identified in the model inventory.

The most relevant parameters for the characterisation of the respiratory tract dosimetry models are summarised in Fig. 4. These include: the MN type used in the model (either to develop or evaluate the model); the species considered to build the model and the input parameters (independent or dependent on MN type) needed to run the model.

#### 3.3.1 Model species for respiratory tract dosimetry models

Assessment of human health risk from exposure to inhaled materials often relies on extrapolation of dose-response data from laboratory animals. Inhalation toxicological studies are mainly conducted in rodents such as mice or rats. Due to the differences in their respiratory tract architecture and other physiological parameters, differences in the lung deposition fraction can occur between test animals and humans. Predicting such lung deposition fractions refine the comparison of doses causing animal toxicity with those associated with human exposure to the inhaled materials.

Three out of six models found in the literature [46], [47] were respiratory tract dosimetry models for rats. Among these, the Multiple-Path Particle Dosimetry (MPPD) model [46] (freely available from https://www.ara.com/products/multiple-path-particle-dosimetry-model-mppd-v-304) has been successively improved by adaptation to mouse, rhesus monkey, pig and rabbit, and has included more specific treatment of the aerosol size through a multimodal size distribution [16]. The MPPD model has been applied quite thoroughly in the field of inhalation hazard assessment [48], [49]. Zhang et al. [50] and Kolanjiyil et al. [26] built their deposition models based on the geometry of the human airway. Finally, Schroeter et al. [51] built a model based on the nasal architecture of the rhesus monkey.

### 3.4 Results of the analysis of available *in vitro* dosimetry models

**Fig. 5:**
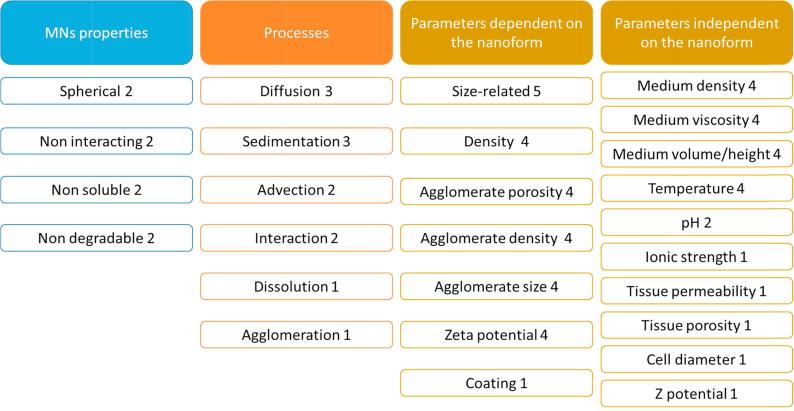
Summary of the type of processes and input parameters (nanoform-dependent, nanoform-independent) identified in the *in vitro* dosimetry models. The numbers represent the times that each specific parameter has been recorded in the model inventory.

Commonly, *in vitro* dosimetry models are based on mathematical approaches that describe the dynamics of particles in liquids. The most relevant parameters in the characterisation of the respiratory tract dosimetry models are summarised in Fig. 5.

Agglomeration plays an important role in *in vitro* dosimetry, influenced by MN concentration, surface chemistry and zeta potential, and affects MNs diffusion and gravitational settling [52]. Surprisingly, agglomeration is recorded only for one model reported in the inventory. Nonetheless, sedimentation (or gravitational settling) is reported in three models in the inventory, according to Fig. 5, as it is an essential process in the fate of MNs [52].

#### 3.4.1 Kinetic processes in *in vitro* dosimetry models applicable to MN

Kinetic processes such as diffusion (based on the Stokes-Einstein equation), sedimentation (based on Stokes law) and advection (transfer by motion of the fluid) are some of the processes that are considered in these models (e.g. *In vitro* Sedimentation, Diffusion and Dosimetry model – ISDD – by Hinderliter et al. [53]). Mukherjee et al. [45] also includes in their agglomeration-diffusion-sedimentation-reaction model (ADSRM), dynamic transformation processes important for MNs, specifically dissolution. Neither the ISDD nor ADSRM models consider the interaction of MNs with molecules present in the test system media. To overcome this limitation in the ASDRM model, the authors [54] extended the ADSRM model to consider the interaction of MNs with various fractions of lipids and surfactant proteins in the model. Another enhancement worth mentioning is the semi-analytical solution for the ISDD model developed by Mahnama et al. [55]. Based on a generalised integral transform technique (GITT) the predictions concerning the advection-diffusion processes were improved, and consequently the accuracy of the ISDD model. The above-mentioned models are mainly designed for *in vitro* supporting systems such as tubes or cell culture well plates.

A different approach was developed to predict the distribution of MNs in the extracellular phase after injection [56]. Interactions of the MNs with the surrounding media were also considered. To this end, van der Waals interactions, electrostatic forces and attachment of MNs to solid structures is taken into consideration as relevant processes in the model.

## 4 Discussion

Information from the PB model inventory reported above allowed description of the status of different models applicable to MNs. We evaluate in this section the relevance of the available models for the assessment of MNs under REACH, also with a view to possible future acceptance of the available model types. We also discuss the model landscape regarding MN coverage and applicability domain, processes taken into consideration, model inputs and outputs, assumptions and uncertainties.

### 4.1 REACH relevance

In general TK data can be used for: “[…] further acceptability and applicability of quantitative structure-activity relationships, read-across or grouping approaches in the safety evaluation of substances. Kinetic data may also be used to evaluate the toxicological relevance of other studies (e.g. *in vivo*/*in vitro*)” (OECD Test Guideline 417 [57]).

REACH allows the use of any scientifically justified information as weight of evidence (Annex XI) supporting read-across approach. As mentioned in the introduction, TK can support the derivation of DNEL from animal data to account for human health risk and to determine AFs: 1) route-to-route; 2) interspecies and 3) high-dose-to-low-dose extrapolation. However, taking into consideration model assumptions, issues related to the applicability domain and model reliability, their application would be associated with high uncertainty.

TK data could aid in the quantification of intraspecies variability, which may be caused by variation in anatomical, physiological and biochemical parameters with, age, gender, genetic predisposition and health status, thus determining specific AFs. Interspecies differences result from variation in the sensitivity of species due to differences in TK and in TD: such models can potentially be used to calculate interspecies assessment factors in the human risk assessment process. However, PBD models are not explicitly taken into consideration in ECHA guidance supporting REACH dossier submissions, although TD contribute to the determination of AFs [7].

Respiratory tract dosimetry models can be used to determine the internal dose following inhalation. However, care must be taken since the most sensitive endpoint may vary for different durations or techniques of exposure, resulting in different internal doses from the same external inhaled concentration. These models can also be applied to extrapolate from animal toxicological data to humans, e.g. calculation of a human equivalent dose (HED) [58].

Finally, *in vitro* dosimetry models can be helpful in IVIVE, and they are mentioned in the draft ECHA guidance on information requirements and chemical safety assessment for estimating biotransformation rates in bioaccumulation assessments [59].

### 4.2 MNs and applicability domain

The concept of applicability domain is important in defining the limits of validity of a quantitative structure-activity relationship (QSAR) (e.g. types of chemical structures, physicochemical properties and mechanisms of action) [60] and is also applied to the PB models in our inventory [18], [61], [62].

Only a few models in the inventory explicitly report the material applicability domain. For instance, Bachler et al. [22] defined the applicability domain as “ionic silver and 15–150 nm nano-Ag, which were not coated with substances designed to prolong the circulatory time (e.g., polyethylene glycol)”. Li et al., [29] stated that the model can be applied to “non-degradable/non-metabolisable nanoparticles”. In most cases, however, the applicability domain is not reported.

In general, dosimetry models reported in the inventory include computational fluid and particle dynamic simulation models that were developed to study airflow, gas uptake and deposition fractions of particles that cover different size ranges (i.e. nanoparticles and microparticles). Hence, this type of model can simulate the behaviour of a “generic” particle but is not limited to particles in the nano-size range. For instance, Schroeter et al. [51], used a CFD model based on the architecture of the nasal passage of an adult and an infant rhesus monkey to simulate inhaled airflow and particle deposition for both inhaled MNs (0.5–1000 nm) and microparticles (1–20 µm). Other authors [46], [47], [50], [63] developed models that can be applied to a generic particle with varying size from 10 nm to 10 µm. Some models were evaluated with particles in the nano-size range. For instance, the MPPD model was applied to MNs inhalation studies [48], [49]; Kolanjiyil et al. [26], compared the predicted nasal depositions with available experimental results using polymeric (polystyrene latex) and metal (silver wool) MNs ranging from 3.6 to 100 nm, for validation purposes. Their study shows that for smaller MNs the deposition to the airways is underestimated by the model; most important parameters in determining the fate of test MNs distribution and deposition are MN size and the airflow rate. These lung deposition studies can be complemented by studies modelling the fate of inhaled particles after deposition onto the pulmonary mucosa [64]. Kirch et al. [64] applied *ex vivo* and computational approaches to investigate the dependency of mucociliary clearance on size, shape, charge and surface chemistry of MNs and microparticles and in their study these parameters did not affect particles penetration in the mucous layer. Polymeric particles (polystyrene particles ranging from 200 nm to 6 µm) and metal oxide particles (maghemite, Fe_2_O_3_ particles ranging from 146 to 555 nm) were investigated.

*In vitro* dosimetry models generally predict the dynamics of particles in liquids or predict the transport of colloids through a porous medium, e.g. [56]. Similar to the respiratory tract dosimetry models, the *in vitro* dosimetry models include, but are not limited to, particles in the range of 0–100 nm. For instance, Hinderliter et al. [53] stated that the ISDD model can be used for non-interacting spherical particles and their agglomerates. The model was tested with multiple sizes of polystyrene spheres (20–1100 nm), 35 nm amorphous silica and large agglomerates of 30 nm iron oxide particles. Other authors [65], [66], [67], [68], using the ISDD, evaluated a variety of different nanomaterials (<100 nm) including metal (Au, Ag) metal oxides (Al_2_O_3_, CeO_2_, CoO, Cr_2_O_3_, Fe_2_O_3_, Fe_3_O_4_, Gd_2_O_3_, Mn_2_O_3_, SiO_2_, TiO_2_, ZrO_2_) and carbon-based MNs.

Similar to the ISDD model, Su et al. [56] assumed that the model can evaluate spherical, chemically inert and solid MNs, the model being applied to 10 nm Fe_3_O_4_ MNs.

Mukherjee et al. [44] evaluated the ADSRM model by applying it to silver-based MNs of different size (from 1 to 110 nm) and surface chemistry (i.e. citrate, PEG and PVP). The model parameters optimised in this exercise can support predictions in *in vivo* inflammatory modelling that would include various cell types and interactions. However, the model can predict the dissolution of silver MNs. In a further publication, the same authors added the assessment of MN interaction with various fractions of lipids and surfactant proteins of the alveolar lining fluid, and applied the model also to 20–110 nm gold MNs and 600 nm SiO_2_ particles [54].

### 4.3 Model inputs and outputs

The most common input parameters in the TK models reported in the inventory are physiological parameters (e.g. body weight, organ weight, blood flow, organ and tissue volumes, blood flow to organs) that are non-dependent on the nanoform. Only one model in the inventory has a size-related input parameter (diameter) [22] that is applied in the calculation of the MPS uptake rate of MNs from blood circulation. Other physicochemical properties such as surface area, specific surface area, density and agglomeration state are not extensively considered as input parameters in the collected models. These physicochemical properties are well known to affect the TK of MNs. However, most available TK models were developed and parameterised on the basis of experimental data for a single MN, and their goal was not to predict TK for other MNs. As other computational kinetic models, the estimation of the concentration in tissues, time-dependent concentration in organs or other specific compartments is the main predicted model outcome of the collected TK models.

The input parameters used in the respiratory tract dosimetry models include some of the physicochemical properties of the MNs. As shown in Fig. 4, two main MN-dependent properties are identified: size related parameters (e.g. diameter, radius and diffusion coefficient) and density. Four groups of nanoform-independent parameters were also identified, related to air properties (e.g. air density, viscosity, flow rate, etc), airway architecture (e.g. airway length, diameter, volume, and area), respiratory function parameters (e.g. tidal volume and breathing frequency) and mucus properties (e.g. thickness and viscosity). The main model output in this case is particle deposition (as mass) in different respiratory sections, e.g. the upper [47], [51], [63], the lower [46] or the whole respiratory tract [26], [50]. The deposited mass can be converted into other dose metrics (such as surface area) if the size, shape and density of the MN are known. In some cases, dose descriptors other than mass have shown better correlation to hazard endpoints [69], [70], [71], so it would be useful if the output of the models were also provided in terms of other dose metrics.

From the analysis of the inventory, MN physicochemical properties are relevant input parameters in the *in vitro* dosimetry models. Size, density and zeta potential of the primary MNs as well as properties related to the agglomeration state (e.g. agglomeration density, size and porosity) are the main parameters reported as nanoform-dependent in Fig. 5. Mukherjee et al. [45] considered the fraction of coated surface as input parameter from which the free MN surface available for reaction is derived. The properties of the assay media are non-nanoform dependent model input parameters, as identified in Fig. 5. *In vitro* dosimetry models collected in our inventory provide as an output the estimation of the time-dependent fraction of administered particles that deposit on cells [45], [53], [54], [55], or the spatial distribution of MNs in the extracellular space [56].

### 4.4 Assumptions and uncertainties

TK models reported in our inventory are based on some relevant assumptions that can be considered as critical factors responsible for part of the uncertainty of the available models. Generally, ideal MNs are taken into consideration when applying a TK model: it is assumed that MN physicochemical properties do not affect their biokinetics, no agglomeration occurs, and, when inhalation is considered as an exposure pathway, there is no overload effect in the lung.

Some assumptions hold also for the model structure and processes:

• All compartments are considered as well mixed (homogeneous), i.e. there are no spatial gradients
• The rates of mechanical transport are independent of the chemical composition and crystal form of the MNs (e.g. [35])
• Although there is some evidence that for MNs the blood:tissue partition coefficient is an important parameter in understanding their fate (e.g. [28]), some studies applied the same blood:tissue partition coefficient value for different organs, to facilitate parameter estimation (e.g. [33])

Respiratory tract dosimetry models assume a uniform concentration of monodisperse MNs [51], and also adopt some simplifications of the airway architecture:

• Homogeneity in the airway geometry (e.g. alveolar volume was assumed to be equally distributed among all alveolar ducts) (e.g. [46])
• Disturbances in the air flow caused by the presence of the nanoparticles are neglected (e.g. [47])
• Constant and homogeneous parameters (e.g. constant velocity of ciliary beating; e.g. [64])

Some examples of assumptions underlying the *in vitro* dosimetry models are:

• Single value for the average particle hydrodynamic diameter (e.g. [53])
• Size, number and effective density of agglomerates remain constant over time (e.g. [53])
• Non-buoyant particles (particles immediately and permanently adhere to cells and are thereby removed from further influencing transport)
• Deposition is assumed to be irreversible (e.g. [56])
• Oxidation of Ag MN coatings is assumed to be zero for PEG and PVP coatings (e.g. [45])

All these assumptions are sources of uncertainty, to different degrees, in the model output. They are not necessarily generalised assumptions in all the reviewed models, but are recognised in at least one of them.

For TD models, declared assumptions and uncertainties are on cellular growth kinetics [41]; in general, the same considerations as for TK models apply regarding nanoform-dependent MN properties as input requirements.

## 5 Conclusions

A number of PB models have been developed and applied to MNs. Particle size is not a required input in all model types: respiratory tract dosimetry models usually have a more detailed consideration of particle behaviour in the airways compared to TK models, which in many cases do not consider any size-related parameter [13], [17], [31], [36]. Regarding the structure of the available PBK models, the assumptions made for the model structure are similar to those made for chemicals (e.g. airway architecture, homogeneous compartments, same blood partitioning coefficients for different organs) [23], [33], [46], [72]. On the other hand, parameters that are specific to MNs like the influence of their physicochemical properties, such as size, on their biokinetics and reversibility of processes like agglomeration and sedimentation are typically neglected as models usually assume that MNs do not agglomerate, and that when inhalation is considered as an exposure pathway, there is no overload effect in the lungs.

Biological systems are complex and the behaviour of MNs is not yet fully understood. The characterisation of MNs in biological contexts calls for analytical and physical experimental techniques combined with computational models, including PBK models, either based on physical principles or employing data-mining strategies [73]. In recent years, research efforts by the scientific community are going in this direction through the creation of tools and platforms for the exchange of information and data and for model creation [19], [20], [74], [75]. These efforts are being strengthened by the ongoing EU project *Grouping, Read-Across, CharacterIsation and classificatiOn framework for regUlatory risk assessment of manufactured nanomaterials and Safer design of nano-enabled products* (GRACIOUS) (Grant Agreement No. 760840), which aims at enforcing data sharing and creating exchange interfaces (https://www.h2020gracious.eu/).

All information relevant for understanding PB model structures and applications are systematically reported in the EURL ECVAM repository on PBK models applied to MNs [19], representing the main outcome of this manuscript. The available inventory was structured to extract those details on the available models that are needed for understanding the specificity of a model to its (regulatory) application, and to facilitate understanding of the reliability of the model results.

Regarding the acceptance and application of PBK models in public health decision making (e.g. in REACH), conclusions drawn by Tan et al. [76] and Paini et al. [77] hold true also for the case of PBK models applied to MNs. There are three main barriers to the more extensive reliance on PBK models for regulatory assessment purposes. One reason lays on the intrinsic complexity of PBK models that makes it challenging to evaluate them. Another aspect relies on the lack of transferability across modelling platforms. To facilitate PBK model code conversion, Lin et al. [78] conducted a study on implementing existing PBK models in different software platforms. One of the two selected PBK models to conduct the comparison was gold MNs. These aspects are not specific to the chemical or material type but are related to the complexity and variety of available PBK models, as well as the difficulty of identifying a representative set of models considered acceptable for regulatory applications. Another issue impacting the acceptance of PBK models in regulatory applications is the lack of confidence in PBK models for chemicals lacking tissue/plasma concentration data [76]. This aspect holds true also for MNs, although in this case there is the additional uncertainty of how such partitioning should be taken into account: while for chemicals the concept of partitioning between tissues and blood is based on the chemical potential of molecules in different phases such as water, fat and protein phase, it is less clear what determines the partitioning of MNs [17]. Thus, there are still barriers to the acceptance of PBK models in regulatory applications for chemicals and these are even greater for MNs, where there is less experience in constructing and parameterising the respective models, and fewer data for model validation purposes. It is expected that the use of standardised reporting formats for PB models, such as those proposed in this study, will help facilitate the dialogue between the developers and proponents of PB models on the one hand, and the users/regulatory assessors on the other.

## Conflict of interest

None.

## Footnotes

1 Physiologically based pharmacokinetic (PBPK) modelling is the most commonly used term for mathematical models of whole organism kinetics, especially in the pharmaceutical field where drugs are simulated. In the context of chemical risk assessment, this is not really appropriate so physiologically based toxicokinetic (PBTK) modelling is often used as well. A more neutral variant is physiologically based biokinetic (PBBK) model. In this paper, for simplicity and consistency, we adopt the general term of physiologically based kinetic (PBK) model for whole organism kinetics. We refer to physiologically based (PB) models to include all the mathematical models applied for interpreting kinetics and dynamics in whole organisms or cells.

2 *S2: Model inventories for physiologically based kinetic (PBK) models, dosimetry models and environmental fate models (Excel workbooks)*.
